# Echocardiography vs. CMR in the Quantification of Chronic Mitral Regurgitation: A Happy Marriage or Stormy Divorce?

**DOI:** 10.3390/jcdd10040150

**Published:** 2023-03-31

**Authors:** Francesca Baessato, Laura Fusini, Manuela Muratori, Gloria Tamborini, Sarah Ghulam Ali, Valentina Mantegazza, Andrea Baggiano, Saima Mushtaq, Mauro Pepi, Giuseppe Patti, Gianluca Pontone

**Affiliations:** 1 Congenital Heart Disease and Pediatric Cardiology, German Heart Center Munich, 80636 Munich, Germany; 2 Department of Cardiology, Regional Hospital S. Maurizio, 39100 Bolzano, Italy; 3 Department of Perioperative Cardiology and Cardiovascular Imaging, Centro Cardiologico Monzino IRCCS, 20138 Milan, Italy; 4 Department of Electronics, Information and Biomedical Engineering, Politecnico di Milano, 20133 Milan, Italy; 5 Department of Translational Medicine, University of Eastern Piedmont, 28100 Novara, Italy; 6 Division of Cardiology, Maggiore della Carità Hospital, 28100 Novara, Italy; 7 Department of Biomedical, Surgical and Dental Sciences, University of Milan, 2012 Milan, Italy

**Keywords:** mitral regurgitation, mitral valve prolapse, echocardiography, cardiovascular magnetic resonance, multimodality imaging, mitral valve surgery

## Abstract

Quantification of chronic mitral regurgitation (MR) is essential to guide patients’ clinical management and define the need and appropriate timing for mitral valve surgery. Echocardiography represents the first-line imaging modality to assess MR and requires an integrative approach based on qualitative, semiquantitative, and quantitative parameters. Of note, quantitative parameters, such as the echocardiographic effective regurgitant orifice area, regurgitant volume (RegV), and regurgitant fraction (RegF), are considered the most reliable indicators of MR severity. In contrast, cardiac magnetic resonance (CMR) has demonstrated high accuracy and good reproducibility in quantifying MR, especially in cases with secondary MR; nonholosystolic, eccentric, and multiple jets; or noncircular regurgitant orifices, where quantification with echocardiography is an issue. No gold standard for MR quantification by noninvasive cardiac imaging has been defined so far. Only a moderate agreement has been shown between echocardiography, either with transthoracic or transesophageal approaches, and CMR in MR quantification, as supported by numerous comparative studies. A higher agreement is evidenced when echocardiographic 3D techniques are used. CMR is superior to echocardiography in the calculation of the RegV, RegF, and ventricular volumes and can provide myocardial tissue characterization. However, echocardiography remains fundamental in the pre-operative anatomical evaluation of the mitral valve and of the subvalvular apparatus. The aim of this review is to explore the accuracy of MR quantification provided by echocardiography and CMR in a head-to-head comparison between the two techniques, with insight into the technical aspects of each imaging modality.

## 1. Introduction

Mitral regurgitation (MR) is the most common valvular heart disease in the United States and the second most common after aortic stenosis in Europe, with an overall prevalence of 2% in the general population [1]. Primary MR is defined by the presence of structural leaflet and subvalvular apparatus alterations and is most frequently caused by mitral valve (MV) prolapse. Secondary or functional MR is a consequence of left ventricular (LV) and/or left atrial (LA) remodeling, dilatation, and dysfunction, which results in tethering and defect of the coaptation of the MV leaflets [2,3].

The entity of MR and its impact on the left heart hemodynamics represent the principal determinants of risk in chronic MR. Over the last years, early cardiac surgery has been increasingly performed in asymptomatic patients with severe MR and has shown excellent outcomes in MV repair and long-term survival [4].

An accurate noninvasive imaging assessment with proper quantification of valve disease covers a central role in the management and surgical planning of patients, especially in discerning severe from nonsevere forms, in the presence of left ventricular dysfunction, and in the absence of overt symptoms. Currently, no gold standard has been established for the definition of the severity of MR by cardiac imaging [4]. The quantification of MR is challenging and needs a structured approach. According to the latest guidelines, the first-line imaging modality is echocardiography, with cardiovascular magnetic resonance (CMR) being recommended in cases where echocardiographic findings are uncertain [5,6].

The aim of this review is to explore the accuracy of MR quantification provided by echocardiography and CMR in a head-to-head comparison between the two techniques, with insight into the technical aspects of each imaging modality. Table 1 illustrates the principal roles and diagnostic accuracy of echocardiographic modalities and CMR to assess chronic MR.

## 2. Echocardiography and CMR Quantitative Parameters: Technical Aspects

The quantification of MR severity is based on the measurement of the EROA and on the entity of volume overload expressed by the RegV and the RegF. The EROA is defined as the mean area of the regurgitant orifice in systole, the RegV is the total amount of blood flow that is regurgitated in systole at each cardiac cycle (mL/beat), and the RegF is a relative measure of the regurgitation given by the ratio of the RegV to the total LV stroke volume (SV) (%) [7,8,9,10].

### 2.1. Echocardiography

The calculation of the quantitative parameters in echocardiography essentially relies on the principles of noncompressibility of blood and mass conservation. For blood flow volume calculation, geometric assumptions are adopted, and the blood flow volume is calculated as the product of the vessel area and the mean velocity of blood [11].

The most used approach for the calculation of the EROA is that derived from PISA [12]. The PISA radius is measured at the flow convergence zone proximal to the regurgitant orifice as evident with color flow imaging. The color Doppler scale baseline is shifted downward in the same direction of the MR jet to measure the PISA radius. The total amount of flow through the convergence zone is assumed to be equivalent to the flow through the regurgitant orifice and is given by the product of the area of the flow convergence hemisphere and the aliasing velocity. The tracing of the continuous wave (CW) Doppler of the regurgitant jet allows for the calculation of the EROA from the ratio of the regurgitant flow to the peak velocity of regurgitation [13]. The RegV is calculated from the product of the EROA with the MR tissue velocity integral (TVI) derived from the MR CW Doppler profile. Another method is to derive the RegV from the difference in the LV outflow tract (LVOT) and mitral SV calculated using the product of the LVOT and mitral annular areas and their integral of forward blood TVI [14,15]. The RegF can then be calculated as the ratio of the RegV to mitral SV. For mitral and aortic SV, it is assumed that the shape of both MV annulus and LVOT is circular. The presence of aortic regurgitation limits this approach [16]. As an alternative, the RegV can be derived from the difference in the LVSV, calculated using a 2D volumetric analysis, and aortic SV [8].

Although quantitative echocardiographic parameters are considered the most reliable methods for quantification of MR, they can be strongly affected by structural and technical factors, such as alterations in valve morphology and its dynamic changes, diffuse calcifications, presence of multiple and eccentric MR jets, imprecise tracking of Doppler profiles, and the need of assumptions for estimations [17,18,19].

Echocardiographic quantification of MR appears to be more challenging for secondary than primary MR. A given value of RegV can become significantly different in relation to the dimensions of the LV and can be smaller in patients with dilated or ischemic cardiopathy, as the LVSV is tendentially reduced, therefore underestimating MR. In the presence of nonholosystolic, biphasic jets, which are commonly seen in secondary MR, the estimation of MR severity can be significantly overestimated by peak-EROA, as it is based on a single frame of measurement [20]. Moreover, small changes in PISA radius measurements can determine large errors in MR quantification [19]. The shape of the regurgitant orifice in secondary MR is often noncircular or elliptical, and in this case, the EROA may underestimate the MR severity. This happens also when multiple or eccentric jets are present.

However, a great advantage of echocardiography is the opportunity to evaluate the dynamic nature of MR characterized by physiological fluctuations occurring in response to various loading conditions (i.e., exercise and ischemia). In both chronic primary and secondary MR, the dynamic behavior of MR can be reliably examined during stress echocardiography. Identifying patients who experience a significant increase in regurgitant volume or in systolic pulmonary artery pressure during exercise provides prognostic insight and risk stratification parameters and, therefore, can help to define the optimal timing of intervention [21].

### 2.2. Cardiovascular Magnetic Resonance

**Figure 1:**
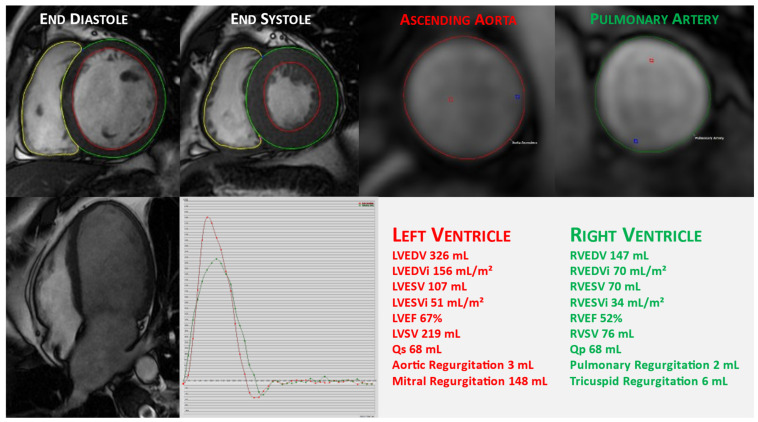
Cardiac magnetic resonance evaluation of mitral valve prolapse with severe mitral regurgitation. The quantification of valvular regurgitation by CMR: evaluation of left (LV) and right ventricular (RV) volumes, ejection fraction, and stroke volume using cine CMR acquisitions. The LV and RV endocardial contours are traced in the diastole and systole from a short-axis stack from the base to apex; aortic and pulmonary flows are assessed using the phase-contrast sequence, and an analysis of the flow time plot is displayed.

The quantification of MR with CMR is based primarily on the performance of two standard sequences: steady-state free precession (SSFP) cine and phase contrast (PC) imaging. The principal method to calculate the MR RegVol with CMR is by the difference in the LVSV derived from the volume quantification on short-axis SSFP images and the aortic flow on PC sequences (Figure 1). In the presence of aortic regurgitation, aortic regurgitant flow must be considered. This method is very reproducible and accurate in the quantification of MR severity (AUC = 0.98), followed by 3D echocardiography (AUC = 0.96), 2D echocardiography (AUC = 0.90), and direct PC on the MV with CMR (AUC = 0.83) [22]. In an alternative to the aortic flow, the RegV can be obtained by subtracting the pulmonary artery flow but not in the presence of cardiac shunts. The RegV can also be derived from the difference in the LV and RV SV or by the difference in the mitral inflow SV and aortic forward SV. This approach cannot be applied in case of multiple valve lesions or intracardiac shunts and is less reproducible.

These methods provide the MR grading independently from the regurgitant jet number and jet direction and without the need for geometric assumptions and contrast agents [23]. The short-axis through-plane PC images on the MV can provide a direct measurement of the RegV but seems less accurate due to the continuous motion of the MV annulus plane during systole [24]. Moreover, as a single PC plane should be planned perpendicular to the MR jet, this may lead to MR underestimation if the jet is eccentric or there are multiple jets [25].

Common errors that can affect SV calculation by CMR may be due to the incorrect segmentation of the LV, poor breath-holding, or arrhythmias. High center expertise is required to obtain reliable data, and routine internal validation studies on volume and flow calculation should be performed to increase the acquisition and analysis quality [26,27]. For flow measurement, a principal limitation is represented by the correct choice of the velocity encoding value, especially in the presence of high-velocity MR jets and concomitant valve stenosis. Baseline phase offset errors due to the fact of inhomogeneity in the magnetic field could be reduced using phantom correction and automated baseline correction tools provided by current commercial analysis software [28]. The acquisition of repeated flows on the same vessel, commonly achieved after three times, is preferable in order to improve the data consistency.

Over the last years, four-dimensional (4D) flow has demonstrated a high accuracy in MR quantification [29,30,31], but it is still confined to the research area. Further studies are warranted to provide a rapid, standardized image analysis for the real application of 4D flow for MR quantification in clinical practice.

## 3. Echocardiography vs. CMR: Comparative Studies and Current Evidence

As no reference standard has been established so far, the difficulty increases in defining the most reliable parameter or imaging approach to quantify the severity of chronic MR. A direct comparison of MR quantitative assessment with CMR and echocardiography has been addressed in different studies involving two-dimensional (2D) transthoracic echocardiography (TTE), in most cases, and, less frequently, three-dimensional (3D) and transesophageal echocardiography (TEE).

### 3.1. 2D Echocardiography vs. CMR

**Figure 2:**
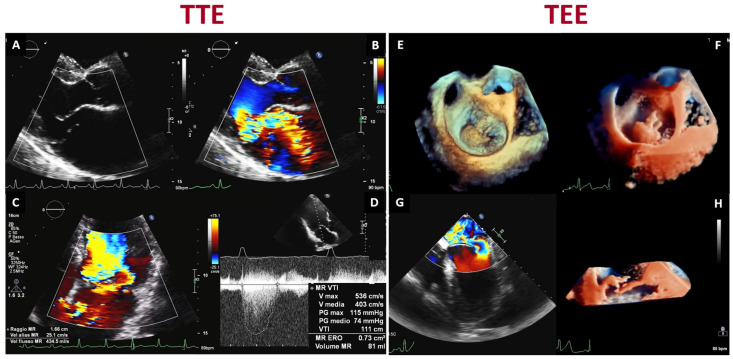
Transthoracic (**left**) and transesophageal (**right**) echocardiographic evaluation of a patient with mitral valve prolapse and severe mitral regurgitation: (**A**) parasternal long-axis view demonstrating a flail of the posterior mitral valve leaflet (scallop P2); (**B**,**C**) assessment of the regurgitant jet by color flow Doppler in parasternal long-axis and apical four chamber views; (**D**) continuous wave Doppler measurement of mitral regurgitation peak velocity and time–velocity integral; (**E**) standard 3D TEE rendering of the mitral valve in a surgical view; (**F**) transillumination rendering technique with a virtual light source highlighting the localization of the chordal rupture; (**G**) 2D color Doppler image of the eccentric regurgitant jet, known as the Coandă effect, hugging the interatrial septum; (**H**) 3D transillumination rendering image cropped at A2-P2 showing the flail of the posterior mitral leaflet.

The significant advantages of echocardiography are its diffuse availability, limited costs, and easy access for all patients. Echocardiography can provide accurate detection of MV structural changes involving the valvular and subvalvular apparatus thanks to its high spatial and temporal resolution (Figure 2).

Hemodynamic features, such as the estimation of pulmonary pressures and modifications during exercise, can also be easily addressed. In contrast, principal concerns of 2D echocardiography intrinsically involve the quantification of MR and its standardized reporting. Only a minority of reports effectively provide real quantitative data, and the MR severity is mostly estimated visually [32]. Moreover, echocardiography needs to integrate multiple parameters that are not necessarily concordant and not always measurable in each patient due to the fact of several geometric and hemodynamic assumptions [8]. In a prospective multicenter study by Uretsky et al., the authors demonstrated a higher accuracy of CMR than 2D echocardiography in 103 patients with chronic MR. Only a moderate correlation between the two modalities (r = 0.6; *p* < 0.0001) was evidenced, which became even lower in cases of severe MR (r = 0.4; *p* = 0.01). Among the patients who underwent MV surgery, only 32% had severe MR as assessed with CMR. In these patients, a high correlation between the amount of pre-operative regurgitant volume (RegV) by CMR and the degree of LV remodeling was observed (r = 0.85; *p* < 0.0001) [33].

A direct comparison between 2D echocardiography and CMR has also been investigated in various single-center studies. Gelfand et al. prospectively defined the thresholds of the regurgitant fraction (RegF) measured by CMR that best correlated with qualitative color Doppler 2D echocardiography in 55 patients with chronic MR. The values with the highest concordance between 2D echocardiography and CMR were ≤15% for mild MR and >48% for severe MR [34]. Cawley et al. prospectively assessed the RegV with TTE and CMR in 26 patients with different grades of MR. Interestingly, the 2D TTE data correlated well with CMR (r = 0.94), and no significant difference for RegV was evidenced but with a different reproducibility of all echocardiographic techniques (range of r = 0.85 to 0.97) [35]. In an important retrospective study, the overall concordance between 2D TTE and CMR was poor in the qualitative assessment and valuable only in cases of mild MR. Concerning quantitative data, there was a modest agreement of the RegV and RegF between TTE and CMR (r = 0.59 and r = 0.54, respectively, *p* < 0.0001), with the most discrepancies being observed in patients with functional MR. The interobserver variability was significantly lower with CMR than with TTE [36].

Interestingly, the most discrepancies between 2D TTE and CMR have been reported in cases of severe MR with high RegV [37,38].

In patients with primary MR, very recently, a prospective, single-center study demonstrated that the presence of flail leaflets and/or the Coandă effect could not be necessarily associated with severe MR when RegV and RegF are calculated with CMR. In this study, MR was graded as severe in only 59% of patients based on RegV CMR and in 40% of patients by means of CMR RegF. However, this was a single-center study in 158 patients, and the analysis was only based on 2D TTE findings, with no 3D or TEE data [39].

Fewer studies have directly compared 2D TEE to CMR data. However, TEE is a semi-invasive modality and is not commonly used for the serial assessment of chronic MR. An absolute modest agreement was reported between CMR and TEE, ranging from 66 to 70% [40], with a significant increase when 3D TEE was performed (97%). In a retrospective, single-center study by Penicka et al., the quantitative assessment of MR with either TTE or TEE was compared to CMR in 258 patients with primary MR. Comprehensively, RegV was significantly higher with echocardiography than with CMR (*p* < 0.05). The lowest agreement was found in cases with multiple jets or late systolic insufficiency (<20%), while the highest agreement was calculated for single, central, and holosystolic jets (90%). In cases of MV disease with eccentric jets, there was a moderate agreement (53%) [41].

### 3.2. 3D Echocardiography vs. CMR

Technological advances have led to the spread of 3D echocardiography to overcome the major limitations of 2D approaches. The main advantages of this technique for MR quantification are its lower dependency on geometric assumptions for the assessment of ventricular volumes and the possibility of 3D visualization of the regurgitant orifice area and color jet. Concerning the anatomical definition of MV structural changes, 3D TTE techniques showed a comparable [42] or even higher [43] accuracy than 2D TEE in the identification of leaflets and scallops involved. 3D TTE allowed for an accurate definition of both simple and complex MV lesions, with a 95% accuracy in the pre-operative assessment [44]. Of note, 3D TEE has shown the highest accuracy in the identification of structural MV alterations (93–99%) [45]. Novel tools such as 3D transillumination have also demonstrated high potential for improving TTE data in terms of the diagnostic accuracy [46].

The application of 3D echocardiography for the evaluation of MR severity has been principally tested against CMR [47]. In a recent meta-analysis, among all echocardiographic approaches, the 3D proximal isovelocity surface area (PISA) agreed best with CMR, with an underestimation of only −3.20 mL (r = 0.84, *p* < 0.001), followed by 3D volumetric methods, which showed an overestimation of 3.73 mL (r = 0.90, *p* < 0.001). In contrast, 2D volumetric analysis showed the worst agreement. In the presence of severe MR, 3D techniques incorrectly estimated the entity of MR in only 14% of patients compared to 2D techniques, which showed inaccuracies in 38% of patients [48].

Comprehensively, the application of 3D echocardiography has shown to be superior to 2D echocardiography in the quantification of MR, with an overall agreement between 49% and 79% in assessing severe MR when compared to CMR. However, some discrepancies still exist when compared to quantitative CMR data [49,50,51]. High operator expertise is required but reproducibility of 3D echocardiography is still an issue.

### 3.3. Prognostic Impact of MR Quantification

The accuracy of MR quantification has also been tested to predict the clinical outcomes and success of MV repair in several studies, mainly by echocardiography. In contrast, the evidence with CMR is still limited.

An increased effective regurgitant orifice area (EROA) ≥ 40 mm^2^ was an independent predictor of mortality and higher cardiac events during follow-up in patients treated medically and prospectively evaluated with echocardiography [52]. Similarly, patients with EROA > 30 mm^2^ and RegV > 45 mL measured by echocardiography showed the highest risk for cardiac death at follow-up [32]. In another prospective study, a vena contracta > 0.4 cm on 2D TTE was associated with a higher rate of adverse cardiac events [53]. In patients with secondary MR due to the fact of ischemic cardiopathy, poor clinical outcomes were found at a smaller EROA (≥20 mm^2^) in contrast to primary MR (≥40 mm^2^) [54].

Concerning CMR, a prospective, multicenter study showed how the RegV calculated with CMR was the best parameter to predict the need for surgery, with an area under the curve (AUC) of 0.81 for a RegV > 55 mL [55]. Similarly, Penicka et al. reported the RegV by CMR as an independent predictor of mortality in patients with chronic MR, with an AUC of 0.72 [41].

In addition to the robust quantification of LV volumes and MR severity, CMR can provide information on tissue characterization. In approximately 20% of cases of primary MR, variable late gadolinium enhancement is reported and has been associated with a poorer postoperative outcome [56] and increased risk of fatal arrhythmias and sudden cardiac death [57,58]. Diffuse fibrosis assessed with parametric mapping sequences may also be present in patients with relevant MR as a sign of myocardial remodeling secondary to volume overload [59]. However, although these data may help to stratify patients’ risk and individualize antiarrhythmic therapy [60], they are not formally included in guidelines to define the need for intervention.

## 4. Application in Clinical Practice

In patients with chronic MR, indication to intervention is provided in cases of severe MR and mainly driven by the presence of clinical symptoms, arrhythmias, LV and LA remodeling, and elevation of pulmonary pressures [6].

**Figure 3:**
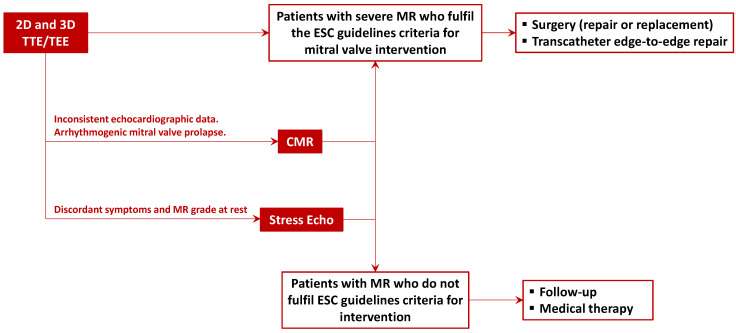
Flowchart of integrated multimodality imaging evaluation of mitral regurgitation.

Therefore, the quantification of MR and LV volumes covers a central role in the indication for surgery. Current 2021 European Society of Cardiology guidelines for valvular heart disease recommend echocardiography in all patients with chronic MR [6]. In cases where MR quantification may be challenging, such as eccentric, multiple, and late-systolic jets or when echocardiographic findings are uncertain, CMR should be performed to add complementary data. At our institution, all patients with at least moderate MR assessed by echocardiography are commonly addressed to CMR to confirm the grading or MR and provide the first volumetric data, as these patients may need seriate follow-up over time (Figure 3).

The identification of the mechanism of lesion is primarily assessed through transthoracic, and eventually transesophageal, echocardiography rather than CMR. In patients with indications to therapy, this is fundamental for targeting the therapeutic approach. Echocardiography, especially 3D TEE, allows for the delineation of MV leaflet anatomy, anular dimensions, and subvalvular apparatus alterations with high accuracy and should be performed in all patients to evaluate the technical feasibility of surgical or percutaneous repair. Surgical repair should be preferred, and percutaneous options, such as transcatheter edge-to-edge repair, should be reserved to patients where clinical and imaging indications for intervention are given but at high risk for surgery. In this context, the performance of CMR may help crosscheck echocardiographic data against criteria for intervention.

## 5. Conclusions

The precise quantification of MR severity is a challenge for a cardiac imager but essential for defining patients’ clinical management and serial follow-up. High center expertise is required for the reliability of both echocardiographic and CMR measurements.

Overall, a significant discordance between echocardiography and CMR has been proved in terms of MR quantification. This discordance is minimized, although not fully eliminated, when 3D echocardiography is used, and maximized in cases of secondary MR, elliptical regurgitant orifices, and eccentric, multiple jets where the evaluation with echocardiography showed the lowest accuracy.

In almost all studies, CMR demonstrated a better reproducibility and lower variability than echocardiography when compared to either 2D or 3D approaches. Moreover, CMR is known to be the gold standard for assessing heart chambers volumes and extension of LV remodeling.

Therefore, when intrinsically concerning absolute MR quantification and calculation of the RegV and RegF, CMR seems to be superior to echocardiography.

However, the prognostic power of quantitative CMR data appears to still be limited in contrast to extensive evidence provided by large echocardiographic studies, and echocardiography remains fundamental and is superior to CMR in the anatomical definition of etiology and underlying mechanism of MR, which is essential in the preprocedural phase of MV repair.

Given the worldwide entity of this clinical phenomenon, further prospective, multicenter, randomized studies should be addressed to provide a larger validated head-to-head comparison of echocardiography against CMR to simplify and optimize the diagnostic path and risk stratification of patients with chronic MR.

## Figures and Tables

**Table 1:** Comparison between echocardiographic approaches and cardiovascular magnetic resonance in the assessment of mitral regurgitation.

	TTE	TEE	CMR
Execution time (minutes) *	20–30	15–20	30–40
Interpretation and reporting time (minutes)	10–15	10–15	15–20
MV leaflet alterations			
Perforation or tear, prolapse, calcification, thickening, and commissural fusion	++	+++	+
Anular diameters	+/++	+++	+++
Mitral anular disjunction	+/++	++	+++
Subvalvular apparatus alterations			
Chordae tendinae: elongation or rupture, thickening or fusion, and tethering	++	+++	+/++
Papillary muscles: elongation or rupture, displacement	++	+++	++
MR quantification			
RegV	++	++	+++
RegF	+	+	+++
EROA	++	++	-
Vena contracta	+++	+++	-
Flow reversal into pulmonary veins	++	+++	+
Number of jets, site, and direction	++	+++	+
LV volumes	++	+/++	+++
Ejection fraction	++	+/++	+++
LA volumes	++	+	+++
Inferior vena cava diameter	+++	++	+++
Inferior vena cava collapsibility	+++	++	+
Pulmonary pressure estimation	+++	++	-

* Does not include time for patient preparation. + = Scale of peculiar abilities. TTE, transthoracic echocardiography; TEE, transesophageal echocardiography; CMR, cardiovascular magnetic resonance; MV, mitral valve; MR, mitral regurgitation; RegV, regurgitant volume; RegF, regurgitant fraction; EROA, effective regurgitant orifice area; LA, left atrial; LV, left ventricular.

## Data Availability

Not applicable.
